# The presence of Auer rods in neutrophils and monocytes besides blasts in a case of acute myeloid leukemia with *CSF3R* mutation

**DOI:** 10.1002/jha2.506

**Published:** 2022-06-16

**Authors:** Haley Koziol, Eileen M. Putnam, Yulei Shen, Juan Gomez‐Gelvez, Kedar Inamdar, Wei Liu

**Affiliations:** ^1^ Touro University Nevada Henderson Nevada USA; ^2^ Department of Pathology and Laboratory Medicine Henry Ford Hospital Detroit Michigan USA

**Keywords:** acute myeloid leukemia, auer rods, CSF3R mutation

1

An 81‐year‐old male with a history of diabetes, hypertension, hypercholesterolemia, previous DVT not on anticoagulation, and arthritis presented to the emergency department with a chief complaint of generalized weakness. Complete blood counts revealed white blood cells (WBC) 165.3 × 10^9^/L, hemoglobin (Hb) 4.4 g/dl, and platelet (PLT) < 10 × 10^9^/L, with 62% blasts. Review of peripheral blood showed blasts of variable sizes with a moderate amount of basophilic cytoplasm (Figure 1A–D; peripheral blood; Leishman stain; 100× objective), and pale‐salmon‐pink‐colored cytoplasmic granules in a subset (Figure 1A, see arrows). Auer rods are easily identified in blasts (Figure 1A,B, see asterisk), occasionally seen in monocytes (Figure 1C, see asterisk) and neutrophils (Figure 1d, see asterisk). Bone marrow was examined to establish diagnosis, showing marked hypercellularity with 72% blasts on aspirate smears with morphologic features similar to those in peripheral blood (Figure 1E [bone marrow biopsy; hematoxylin and eosin, 40× objective] and F [bone marrow aspirate; Leishman stain; 50× objective]). Granulocytic dysplasia with pelgeroid morphology and abnormal nuclear chromatin clumping was seen in both peripheral blood and bone marrow (> 10% and < 50%). Concurrent flow cytometry showed increased myeloblasts with expression of CD34, CD13, CD 117, CD33, HLA‐DR, CD19 (dim; minor subset), CD11b (dim; subset), CD14 (dim; minor subset), CD15 (dim; minor subset), myeloperoxidase (dim; subset), and terminal deoxynucleotidyl transferase (dim; minor subset). Conventional cytogenetics showed normal male karyotype. Concurrent FISH studies using acute myeloid leukemia (AML) and myelodysplastic syndrome panels of probes are negative for all loci studied, including 8q22 and 21q22 probes for *RUNX1‐RUNX1T1* rearrangement. Next generation sequencing showed mutation on the *CSF3R* gene (p.T640N, variant allele frequency 48.4%). An integrated diagnosis of AML with maturation was established (French–American–British Classification Subtype M2).

**FIGURE 1:**
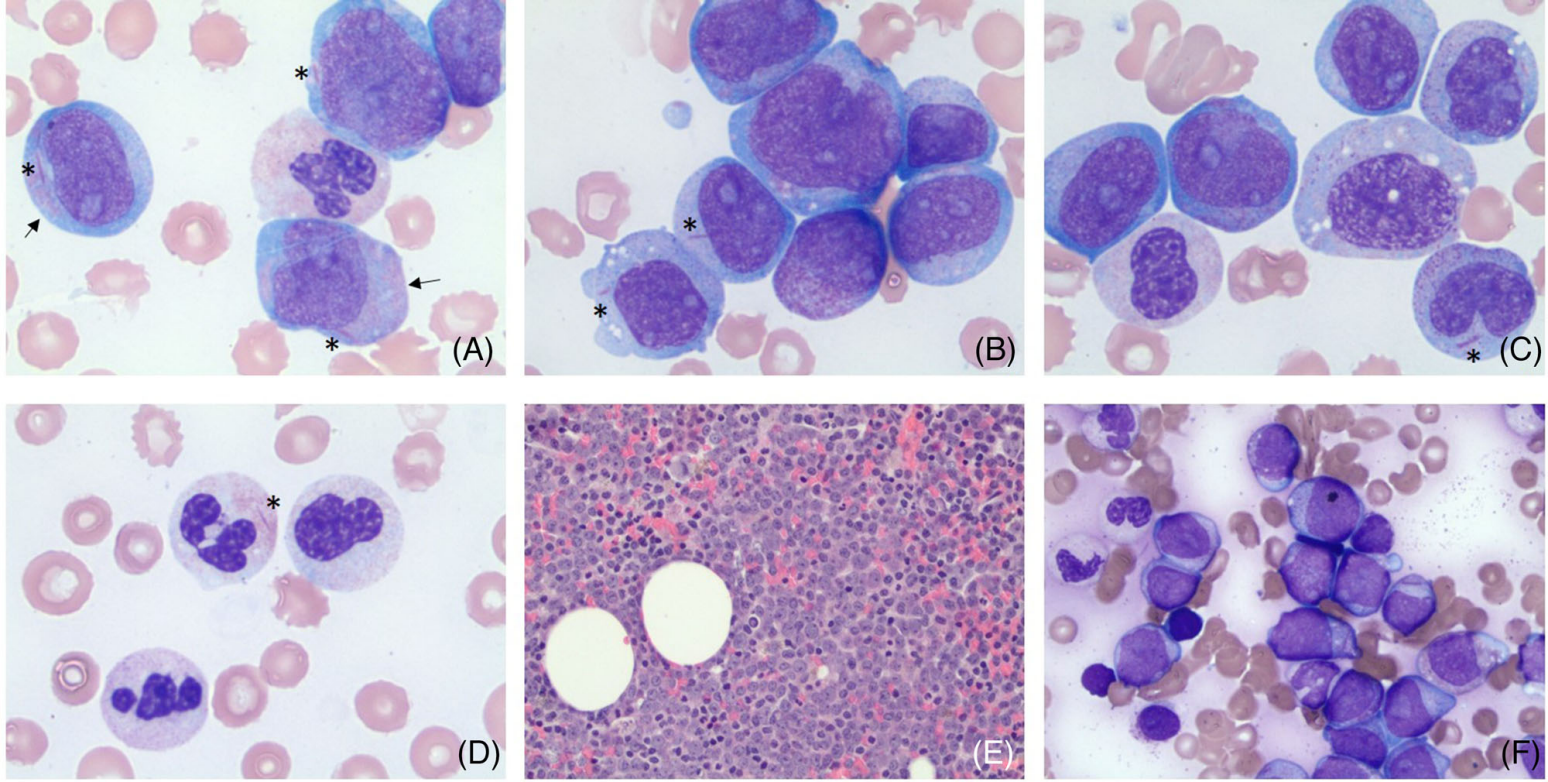
Auer rods in blasts, neutrophils and monocytes in a case of acute myeloid leukemia (AML) with *CSF3R* mutation

Auer rods are a morphologic feature in leukemic blasts defining myeloid differentiation. The presence of Auer rods in neutrophils and monocytes are rarely reported, most commonly in association with acute promyelocytic leukemia (APL) with *PML‐RARA* and AML with *RUNX1‐RUNX1T1*, occasionally seen in other subtypes of AML and myelodysplastic syndrome (MDS) in the literature as well [1]. We presented a case of AML with Auer rods in non‐blast cells as well as morphologic and immunophenotypic features resembling AML with *RUNX1‐RUNX1T1*: blasts with basophilic cytoplasm, salmon‐pink (pale) cytoplasmic granules and perinuclear Hof besides Auer rods as well as granulocytic dysplasia in the background. However, no recurrent cytogenetic and molecular genetic abnormalities as defined by World Health Organization (WHO) classification are detected, while a *CSF3R* mutation seen. Although mutations in the colony stimulating factor 3 receptor (*CSF3R*) occurs rarely in AML, comprising only about 1% of adults with AML, it is suggested that *CSF3R* can be a potential therapeutic target in AML [2].

In summary, we presented for the first time a case of AML with a few rare morphologic features including the presence of Auer rods in non‐blast cells coinciding with a potential therapeutic target of *CSF3R* mutation. Whether the association between the morphologic features and the *CSF3R* mutation can be established warrants additional studies. In addition, a potential diagnostic pitfall to be taken into consideration in this case is that no reverse transcription‐polymerase chain reaction (RT‐PCR) was done to further exclude *RUNX1‐RUNX1T1* rearrangement, although the likelihood of having undetected rearrangement by karyotyping and FISH studies is extremely low.

**Novelty Statements**

1. What is the new aspect of your work?
  Here, we for the first time presented a case of AML with morphologic features resembling AML with *RUNX1‐RUNX1T1*, including Auer rods in non‐blast cells, coinciding with *CSF3R* mutation, which is a rare mutation in AML while representing a potential novel therapeutic target.
2. What is the central finding of your work?
  A rare case of *CSF3R*‐mutated AML with no recurrent cytogenetic abnormality, showing unusual morphologic features resembling AML with *RUNX1‐RUNX1T1*, including Auer rods in non‐blast cells.
3. What is (or could be) the specific clinical relevance of your work?
  The rare morphological features present in this AML could potentially serve as a trigger to identify cases of AML with *CSF3R* mutation for targeted therapy.

## CONFLICT OF INTEREST

The authors declare that there is no conflict of interest that could be perceived as prejudicing the impartiality of the research reported.

## FUNDING INFORMATION

The authors received no specific funding for this work.

## ETHICS STATEMENT

Not applicable.

## PATIENT CONSENT STATEMENT

Not applicable.

## PERMISSION TO REPRODUCE MATERIAL FROM OTHER SOURCES

Not applicable.

## Data Availability

The data that support the findings of this study are available from the corresponding author upon reasonable request.
